# A Heuristic for Disassembly Planning in Remanufacturing System

**DOI:** 10.1155/2014/949527

**Published:** 2014-04-23

**Authors:** Jinmo Sung, Bongju Jeong

**Affiliations:** Department of Information & Industrial Engineering, Yonsei University, 50 Yonsei-ro, Sinchon-dong, Seoul 120-749, Republic of Korea

## Abstract

This study aims to improve the efficiency of disassembly planning in remanufacturing environment. Even though disassembly processes are considered as the reverse of the corresponding assembly processes, under some technological and management constraints the feasible and efficient disassembly planning can be achieved by only well-designed algorithms. In this paper, we propose a heuristic for disassembly planning with the existence of disassembled part/subassembly demands. A mathematical model is formulated for solving this problem to determine the sequence and quantity of disassembly operations to minimize the disassembly costs under sequence-dependent setup and capacity constraints. The disassembly costs consist of the setup cost, part inventory holding cost, disassembly processing cost, and purchasing cost that resulted from unsatisfied demand. A simple but efficient heuristic algorithm is proposed to improve the quality of solution and computational efficiency. The main idea of heuristic is to divide the planning horizon into the smaller planning windows and improve the computational efficiency without much loss of solution quality. Performances of the heuristic are investigated through the computational experiments.

## 1. Introduction

**Figure 1:**
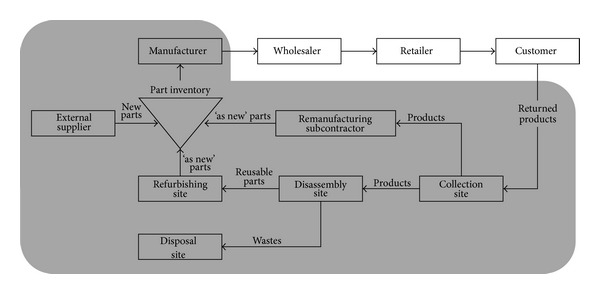
Conceptual framework of remanufacturing [22].

In a recent decade, most of environment-conscious industries have recognized the importance of remanufacturing where end-of-life products are collected and some useful parts are used again to remanufacture new parts. Remanufacturing may save resources on earth and production costs of company. As shown in Figure 1, end-of-life products are processed in remanufacturer to produce “as new” parts or disassembled to extract reusable parts which are furbished and used again by manufacturer.

In this paper, we deal with disassembly processes and especially focus on disassembly planning. Because disassembly is a preprocess of refurbishing or recycling, efficiency of it affects the whole remanufacturing system. In sustainable and environment-conscious industry, as the number of returned products increases, disassembly process becomes more significant and its related decision-making is getting complicated for most companies.

## 2. Related Literatures

As though disassembly planning problem in remanufacturing system looks like just the reverse of the conventional assembly planning problem, it is quite different in the purpose of retrieving necessary parts with the choice of efficient disassembly operations, which requires some different and unique approaches. Also, it deals with not only necessary parts but also unnecessary parts that are disposed or used later in future purpose. With all these reasons, disassembly planning problems is generally considered to be more complicated than assembly planning.

Previous researches on disassembly planning can be classified into three categories: full disassembly of a product with efficient sequence, disassembly to maximize the value of disposal parts, and disassembly to retrieve specific parts with minimum costs. The first category research aims to generate efficient or even optimal sequence of disassembly operations to disassemble a whole product. Most researches try to minimize disassembling costs because the costs are dependent on the selection of disassembly sequence. As one of the corner stone researches, Homem de Mello and Sanderson [1, 2] proposed a basic representation scheme of disassembly sequences with AND/OR graph, though it does not provide an optimal disassembly sequence. Lambert [3] proposed an approach to determine the optimal disassembly sequence using AND/OR graph scheme. He used Bellman's dynamic programming to select disassembly operations at each disassembly stage in backward achieving the minimum costs of whole disassembly costs. These researches have the limitation of application in remanufacturing system where only useful parts need to be obtained.

The second category of research focuses on maximizing the value of disassembled parts, which is very practical issue in remanufacturing system. The problem is to maximize total profit incurred by disassembly process by comparing the costs of disassembly to the value of all disassembled parts. Gupta and Taleb [4] emphasized that the purpose of disassembly process is not to manufacture as new products, but to meet the demand of useful parts, and they proposed a disassembly planning approach using MRP. Go et al. [5] reviewed many disassembly methods to enhance the recovery of end-of-life products, especially vehicle, so that valuable parts are efficiently retrieved. Lambert [6–8] proposed a mathematical model to find the optimal disassembly sequence with sequence-dependent setup costs using AND/OR graph representation. In order to solve this NP-hard problem, he employed iterative method which solves the relaxed problem without precedence constraints, checks the feasibility with added constraints, and then solves the problem with unsatisfied constraints in iterative manner. Although Lambert's method was proven to be very efficient in finding an optimal solution, it assumed only single planning period, single assembly product, and no demand for reusable parts. The last category is literally disassembly planning in remanufacturing environment, which means that it determines disassembly sequence and the amount of disassembly operations with minimum costs. Barba-Gutiérrez et al. [9] developed Reverse MRP using lot-sizing technique and analyzed the impact of lot-sizing technique on disassembly costs. Veerakamolmal and Gupta [10, 11] studied the effect of end-of-life disassembly by evaluating the design efficiency. They used a disassembly tree (DT) structure to represent the precedence relationships which show the order of disassembly parts. The major advantage of DT structure is to retrieve necessary parts at each branch point and make it possible to get demand of disassembly parts. However, as the number of parts in a product, the size of tree increases so rapidly. Furthermore, they [12] solved selective part recovery planning for electronic products using two-stage disassembly and retrieval system, even though the recovery planning is inapplicable to determine the schedule of disassembly operations. More recent researches consider disassembly planning over multiple periods [13–15], where the precedence among disassembly operations is constrained by part inventory and the schedule of disassembly operations is determined according to inventory costs and setup costs to meet the demand of parts. In these researches, however, since the disassembly operations are constrained by the amount of operations, not time constraints, the application to real environment may be very limited without adjustments.

For development of heuristic in this paper, we consider the rolling horizon planning technique which was firstly proposed by Modigliani and Hohn [16]; thereafter abundant applications followed. Chand et al. [17] emphasized the usage of the rolling horizon techniques including forecast and solution procedure in real operation problems based on time-series. They classified the previous operation planning researches by horizon type, model type, source of horizon, solution method, and subject and reviewed the techniques. Schwarz [18] used the rolling horizon planning technique to solve facility location problem, and other researchers such as Blackburn and Millen [19], Chand [20], and Federgruen and Tzur [21] applied it for inventory management problem. Most researches took advantage of rolling horizon technique in planning itself purpose. This study also employed the rolling horizon technique, but for improving the efficiency of solution procedure instead of planning scheme.

The previous researches show that disassembly planning is more than just the reverse of assembly planning but should consider the recovery of useful parts/materials, impact of disposal on environment, and total costs as well. In this study, we are concerned about disassembly planning with efficiency and applicability in real remanufacturing environment. In order to do that, we make disassembly planning allow time-constrained operations, sequence-dependent setup, disassembled part demand, and multiple planning periods, and finally we propose an efficient heuristic to solve this NP-hard problem. This paper is organized as follows: We first develop a mathematical model of disassembly planning in Section 2 and propose a heuristic using rolling plan scheme in the following section. Computational experiments are performed using an example product to show the validity of our planning system and concluding remarks and discussion follow in Section 6.

## 3. Mathematical Model for Disassembly Planning

Disassembly planning in this study has the objective to minimize total disassembly costs of meeting part demands at each period. We recognize that this problem has the same property of the capacitated lot-sizing problem. Moreover, disassembly planning in remanufacturing has the demands of subassemblies at each period, not only the end parts in BOM (Bill-of-Material) structure. Also, since most workshops with disassembly process belong to small- or medium-size company which produces so many different types of products, we assume job-shop production environment rather than mass production line. This means that setup time and the related costs need to be included in the model. For the purpose of modeling, we assume the following production environment.

**Figure 2:**
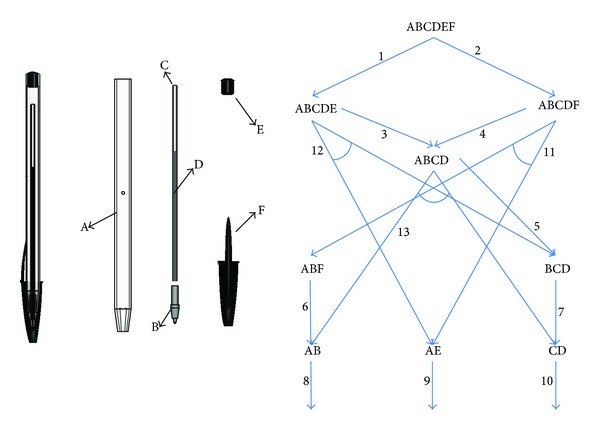
Disassembly AND/OR diagram of ball-pen [23].

- The demands of parts/subassemblies are known at each period.
- The unfulfilled demand through disassembly is fulfilled by purchase from vendor. The purchase cost is considered as penalty cost.
- All setup and workload can be transformed to time units.
- There exists a working available time at each production period.
- Setup can be carried over the next period, that is, the same operation can be carried over the next production period.
- Transition matrix: *T* is a transition matrix with all disassembly operations (column) and subassemblies (row). The elements of *T* are interpreted as follows. For example in Table 1, disassembly operation 7 decomposes the subassembly BCD into CD and B with the values −1 (destruction) and 1 (creation) in the corresponding elements. Table 1 represents a transition matrix corresponding to a ball-pen of Figure 2.

With the above assumptions, the nomenclature shown at the end of the paper is needed for our model.

**Pseudocode 1:**
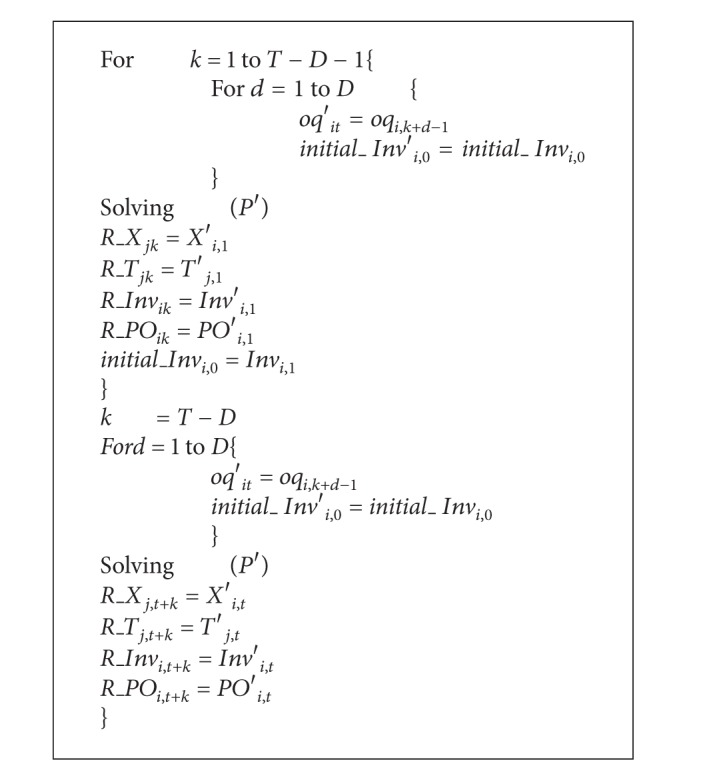


The objective function is represented as the sum of all relevant costs such as operation processing cost, inventory holding cost, setup cost, and purchasing cost. Then the model for disassembly planning is formulated as follows.

Model (*P*)

(1) Min⁡∑j(pcj×∑tXjt)+∑i(hci×∑tInvit)  +∑j(scj×∑tZjt)+∑i∑tppi×Poit

subject to

(2) Invit=Invi,t−1−∑jpsij×(prj×Xjt) +∑jcsij×(prj×Xjt)+Poit−oqit ∀j,t

(3) $$\begin{array}{c} p_{j}\times X_{jt}\leq \underset{j}{\sum }{\text{ps}}_{ij}\times {\text{Inv}}_{i,t-1}\;\forall j,t \end{array}$$

(4) $$\begin{array}{c} \underset{j}{\sum }{\text{st}}_{j}\times Z_{jt}+\underset{j}{\sum }X_{jt}\leq {\text{timeCapa}}_{t}\;\forall t \end{array}$$

(5) $$\begin{array}{c} \underset{j}{\sum }A_{jt}=1\;\forall t \end{array}$$

(6) $$\begin{array}{c} \underset{j}{\sum }B_{jt}=1\;\forall t \end{array}$$

(7) $$\begin{array}{c} X_{jt}\leq M\times Z_{jt}\;\forall j,t \end{array}$$

(8) $$\begin{array}{c} A_{jt}\leq Y_{jt}+W_{t}\;\forall j,t \end{array}$$

(9) $$\begin{array}{c} B_{jt}\leq Y_{jt}+W_{t}\;\forall j,t \end{array}$$

(10) $$\begin{array}{c} A_{jt}-B_{j,t-1}\leq Z_{jt}\;\forall j,t \end{array}$$

(11) $$\begin{array}{c} Y_{jt}-A_{jt}\leq Z_{jt}\;\forall j,t \end{array}$$

(12) $$\begin{array}{c} B_{j,t-1}+W_{t}\leq B_{jt}+1\;\forall j,t \end{array}$$

(13) $$\begin{array}{c} B_{j,t-1}+W_{t}\leq A_{jt}+1\;\forall j,t \end{array}$$

(14) $$\begin{array}{c} \underset{j}{\sum }X_{jt}\leq 1-W_{t}\;\forall t \end{array}$$

(15) $$\begin{array}{c} \underset{j}{\sum }Y_{jt}\leq 1-W_{t}\;\forall t \end{array}$$

(16) $$\begin{array}{c} \underset{j}{\sum }Y_{jt}-1\geq \left(N-1\right)\times \delta_{t}\;\forall t \end{array}$$

(17) $$\begin{array}{c} A_{jt}+B_{jt}\leq 2-\delta_{t}\;\forall j,t \end{array}$$

(18) $$\begin{array}{c} Y_{jt},Z_{jt},A_{jt},B_{jt},W_{t}\in \left\{0,1\right\}\;\forall j,t \end{array}$$

(19) $$\begin{array}{c} {\text{Po}}_{it}\geq 0,\;{\text{Inv}}_{it}\geq 0\;\forall i,t \end{array}$$

(20) $$\begin{array}{c} X_{jt}\geq 0\;\forall j,t \end{array}$$

(21) $$\begin{array}{c} \delta_{t}\geq 0\;\forall t. \end{array}$$

Constraints (2)–(4) ensure the sequence of subassembly operations to be performed. Constraint (2) is the balance equation of inventory between two consecutive periods. Constraint (3) ensures the precedence relationship among subassembly operations with regard to inventory of subassembly in each period. Constraint (4) imposes the total capacity limit in each period. Constraints (5)–(17) represent the requirements and conditions for operations and setups to be carried over the whole planning horizon. Constraints (5) and (6) indicate that each period should have the starting and finishing operations, respectively. Constraint (7) guarantees that disassembly operation without setup is not allowed over all periods. Constraints (8) and (9) ensure the feasible relationship among *A*, *B*, *Y*, and *W* variables. Constraints (10) and (11) indicate that over the planning horizon if the same operation is carried over the next period, there should be no setup. Constraints (12) and (13) represent that if there is no operation performed in the current period, the setup is already done in the previous period so that no setup is necessary in the current period. Constraints (14) and (15) guarantee that the indicator variables of operations should have the valid values accordingly. Finally, constraint (16) and (17) ensure that if only one operation is performed in a period, it means that the first and last operation should be the same operation. The last set of constraints (18)–(21) represent the nonnegativity and binary conditions of all decision variables used in the model.

## 4. Heuristic

### 4.1. Basic Concept

**Figure 3:**
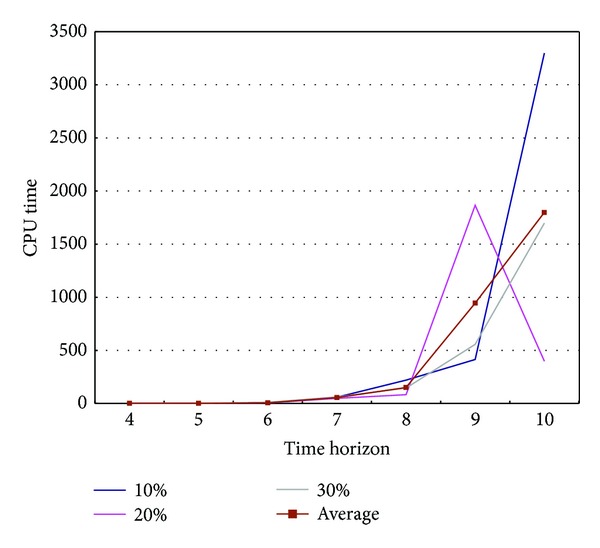
Computation time (sec) according to the length of planning horizon and demand quantity.

In order to develop a heuristic for the problem presented in the previous section, we first examined the computation time for finding the optimal solution. Computational experiments were done using OPL Studio 6.0 by ILOG on the Pentium 4 2.8 Ghz Win-XP platform. We generated 21 types of problems by changing the size of demand (three types) and the length of planning horizon (seven types). Demand sizes are determined by the proportion of production capacity, that is, the order quantity of 10%, 20%, and 30% of production capacity, and the planning horizons are in the range of 4 to 10 planning periods, that is, total 6 types of planning horizon. For each type of problem, ten problems are generated using random number generator and total 210 problems were finally tested.  Figure 3 shows the average computation time taken to solve each type of problem. The result shows no regular pattern according to the size of demand. However, increasing the length of planning horizon seems to make the computation time also increase. In particular, the computation time increases rapidly from planning horizon 7, while it stays very short until then.

**Figure 4:**
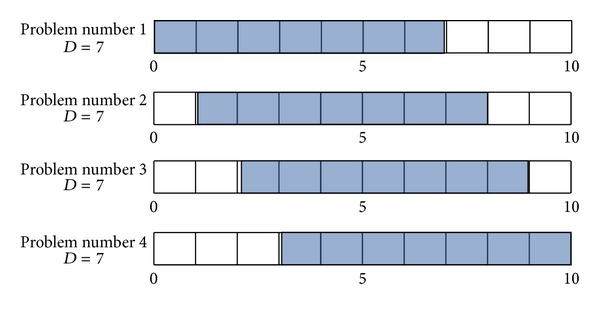
Generation of subproblems using rolling horizon technique.

The investigation indicates that the computation time for solving the mathematical model increases in highly nonlinear fashion as the length of planning horizon increases, but there seems to be no strong relationship with demand pattern. Based on the experiments, in order to overcome the problem with the long planning horizon in real production environment, we consider decomposing the original problem into the smaller size of subproblems with shorter planning horizon and then aggregating them back to get the final solution.  Figure 4 illustrates an example of decomposition. For example, the original 10-periods problem is decomposed into four 7-periods subproblems where each subproblem shifts the planning horizon by one period until the last period is reached. Moreover, the modified cost parameters are adapted to make reasonable global solution. Thus, instead of solving one large problem, we solve four smaller problems separately with efficient time and integrate four solutions to make a solution for the original problem.

This procedure is known to be rolling horizon planning technique which was firstly proposed by Modigliani and Hohn [16]. However, since the subproblems are treated as an independent local problem, there should be a remedy to overcome the local optimality. In order to achieve global optimality for the original problem, we need to adjust setup and inventory holding costs for the local problem so that the local solution can be a part of global solution in the later aggregation procedure. This is because, basically, our model pursues the optimal trade-off between setup and holding costs. For example, if setup cost is much greater than holding cost in the original problem, we need to keep the number of setups small and the inventory as long as possible over the planning horizon. However, since the subproblem with short planning horizon cannot keep inventory for future demand, we adjust the setup cost to be smaller so that more operations are flexibly assigned for future demand. After solving all subproblems, the local solutions are aggregated to be global solution for the original problem.

### 4.2. Heuristic

**Figure 5:**
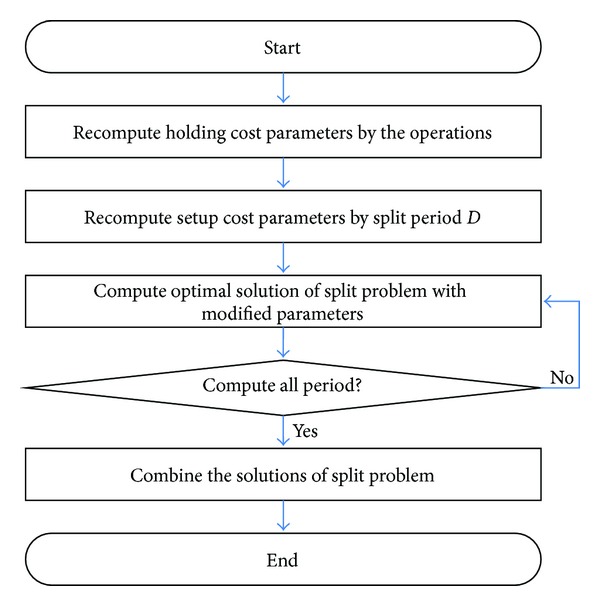
Procedure of heuristic.

Based on the concept described before, our heuristic consists of three steps: (1) adjusting setup cost, (2) solving subproblems, and (3) aggregation of solutions.  Figure 5 presents the whole procedure of heuristic.

#### 4.2.1. Adjusting Setup Cost

Setup costs for each subproblem are adjusted as follows.

(i) Set a fixed planning horizon of subproblem, such as *D* < *T*. In order to improve the quality of solution,* D* needs to be greater than the maximum number of operations for complete disassembly because the precedence relationship among subassembly operations is constrained by inventory of subassembly in each period as indicated in constraint (3) of our mathematical model.
(ii) Inventory holding cost of subassembly (hc_*i*_) is transformed to operation-based cost (hc_*j*_′) as follows:
  (22) $$\begin{array}{c} {\text{hc}}_{\left(1\times j\right)}^{\prime }={\text{hc}}_{\left(1\times i\right)}\times {\text{rm}}_{\left(i\times j\right)}. \end{array}$$
(iii) New inventory cost (hc_*j*_′) represents the changed holding cost by operation *j*. In other words, it is the difference between inventory holding cost before and after subassembly operation is performed.
(iv) Then, new setup cost (sc_*j*_′) is computed as follows:
 Case 1: If sc/hc′ < *D*, sc′ = sc,
 Case 2: If sc/hc′ > *D*, sc′ = hc′ × *D*,
 Case 3: If hc′ = 0, sc′ = 1.

#### 4.2.2. Solving Subproblems

Using adjusted setup cost, each subproblem with planning horizon *D* is solved independently. For the feasibility of global solution, we need an additional constraint (23) as follows, which links all subproblems.

(23) $$\begin{array}{c} {\text{Inv}}_{i,0}={\text{initial}\_\text{Inv}}_{i}\;\forall i. \end{array}$$

Then the model (*P*) is modified to the subproblem models (*P*′) with the additional constraint (23) and adjusted setup costs. The solution procedure for model (*P*′) using rolling horizon technique is presented as follows.

Set iteration, *n* = 1.

- The demand quantity for the *n*th subproblem is determined by the proportional quantity of total demand until period *D*.
- Solve the problem *P*′(*n*). If *P*′(*n*) is the last subproblem, stop. Aggregate the solutions at the first period of each subproblem to make a global solution.
- The inventory at the first period of *P*′(*n*) becomes initial inventory of the next subproblem, *P*′(*n* + 1).
- Set *n* ← *n* + 1. Go to (i).

#### 4.2.3. Aggregation of Solutions

Aggregation of subproblem solutions is simple. All the solutions are represented as the cost parameters of the original problem, but decision variables are replaced with the aggregated solution of subproblems as follows:

(24) ∑j(pcj×∑tRXjt)+∑i(hci×∑tRInvit)  +∑j(scj×∑tR_Zjt)+∑i∑tppi×R_Poit.

The heuristic is programmed with pseudocode as shown in Pseudocode 1.

In the pseudocode, *R*_*XX* is solution for global solution and *XX*′ is for subproblems.

## 5. Computational Experiments

In this section, the efficiency of our heuristic is investigated through computational experiments with the ball-pen example in Figure 2.  Table 2 shows the data of unit holding cost of subassemblies and setup cost of operations. The various production environments are reflected by using cost structure and ease of disassembly. We consider two cost structures according to the relative percentage of processing cost out of setup cost as around 10% (Case A) and 40% (Case B). We assume that there are two types of products based on ease of disassembly, easy (product V) or difficult one (product W).  Table 3 shows the unit processing cost of disassembly operations by Cases A and B, and Table 4 provides the unit purchase cost of subassemblies. In Table 5, production rate and setup time of operations are given.

All experiments were implemented using OPL Studio 6.0 by ILOG on the Pentium 4 2.8 Ghz Win-XP platform. For efficiency of experiments, we limited the run time at 5,400 seconds and over the limit the best value was taken. We considered three different planning horizons of 10, 15, and 20 periods. Demand data were generated according to the ordering probability of 10, 20, and 30%. For each ordering probability, two sets of data were generated. Therefore, 18 experiments (=6 types of demand ∗ 3 planning horizons) were performed for each cost structure (AV, AW, BV, and BW). The results are shown in Table 6 in terms of computation time and the quality of solution. The number of solution indicates the number of optimal solutions found by our heuristic out of 18 experiments. “Optimal” indicates the comparative results to the optimal solutions by OPL and “All” means the comparative performance to all solutions by OPL whether optimal or not.

From the results, we observe that the proposed heuristic finds a solution with very short computation time and the quality of solution is also quite good with less than 5% deviation from the optimal solution. We also find many cases where as the planning horizons increase the accuracy of solution is deteriorated. In order to improve it, one method is to make the subproblems with the longer planning horizon (*D*).  Table 7 illustrates the results of this idea. For the 10-periods planning problem, solving the subproblems with 7-periods horizon provides the better cost results than 6-periods horizon subproblems. However, the increase of planning horizon results in the increase of computation time so rapidly. Thus determining the length of planning horizon for subproblems is the matter of trade-off between the accuracy and time efficiency.

In summary of experiments, our heuristic is quite good enough to be used in real disassembly planning system because of the high quality of solution and efficient computation time.

## 6. Conclusions and Discussion

Disassembly planning determines the sequence and schedule of disassembly operations to meet demand of disassembled subassemblies in remanufacturing environment. Unlike assembly planning, it is much more complicated due to reverse sequence generation and demand of in-process subassemblies. However, most previous researches have certain limits in applying to multiperiods disassembly planning with demand.

In this study, we formulated disassembly planning problem over multiperiods planning horizon with demands of subassemblies. Since the problem is known to be NP-hard, it requires tremendous computational time for reasonable size of problems in real production environments. For practical solution methods, we developed a heuristic based on rolling horizon planning technique which is useful in decomposing the large size problem and solving the subproblems efficiently. The heuristics has been investigated through computational experiments on a set of data reflecting various disassembly planning environments. The results show that our heuristic can be a good alternative method applicable to real production system in terms of computational efficiency and the quality of solution.

Remanufacturing system is still open area to be investigated and improved, especially for identifying many practical issues regarding sustainable environment. We need to continuously find those issues and provide good methods. Specifically, the concept behind the proposed heuristic in this study can be applied to many planning problems found in remanufacturing system in order to improve the planning efficiency.

## Figures and Tables

**Table 1:** Disassembly transform matrix of ball-pen with 15 subassemblies.

	1	2	3	4	5	6	7	8	9	10	11	12	13
ABCDEF	−1	−1											
ABCDE	1		−1									−1	
ABCDF		1		−1							−1		
ABCD				1	−1								−1
ABF						−1					1		
BCD					1		−1					1	
AB						1		−1					1
AE									−1			1	
CD							1			−1	1		1
A								1	1				
B							1	1					
C										1			
D										1			
E		1	1						1				
F	1			1		1							

**Table:** (a)

Subassembly	ABCDEF	ABCDE	ABCDF	ABCD	ABF	BCD	AB	AE	CD	A	B	C	D	E	F
hc_*i*_	0	9	8	6	6	8	5	6	5	4	4	2	1	1	1

**Table:** (b)

Operation	1	2	3	4	5	6	7	8	9	10	11	12	13
sc_*j*_	50	40	30	20	10	50	40	30	20	10	50	40	30

**Table 3:** Unit processing cost of operations (pc_*j*_).

pc_*j*_	1	2	3	4	5	6	7	8	9	10	11	12	13
A	5	4	3	2	1	5	4	3	2	1	5	4	3
B	20	16	12	8	4	20	16	12	8	4	20	16	12

**Table 4:** Unit purchase cost of subassemblies (pp_*i*_).

pp_*i*_	ABCDEF	ABCDE	ABCDF	ABCD	ABF	BCD	AB	AE	CD	A	B	C	D	E	F
A	0	58	56	52	46	44	42	40	38	34	32	30	28	26	24
B	0	174	168	156	138	132	126	120	114	102	96	90	84	78	72

**Table 5:** Production rate (pr_*j*_) and setup time of operations (st_*j*_).

Operation		1	2	3	4	5	6	7	8	9	10	11	12	13
V	pr_*j*_	50	25	100	25	75	50	25	20	25	10	50	100	25
V	st_*j*_	0.15	0.05	0.10	0.20	0.15	0.05	0.15	0.05	0.01	0.05	0.10	0.15	0.15
W	pr_*j*_	40	25	25	40	60	70	45	40	100	90	55	70	65
W	st_*j*_	0.20	0.15	0.10	0.20	0.10	0.20	0.05	0.20	0.15	0.15	0.20	0.05	0.05

**Table 6:** Performance results of heuristic.

Cost structure	Number of optimal solution	Avgerage computation time (sec)				Deviation from OPL solution			
		OPL		Heuristic		*D* = 6		*D* = 7	
		Average optimal	Average total	*D* = 6	*D* = 7	Optimal	All	Optimal	All
A, V	7	1183.9	Over 6983.1	57.8	156.6	0.96%	3.32%	0.74%	2.45%
A, W	6	675.1	Over 7223.9	107.5	252.8	3.00%	2.09%	3.04%	1.73%
B, V	7	916.5	Over 23246.9	95.2	376.7	2.30%	4.39%	1.44%	3.25%
B, W	10	20653	Over 27045	185.9	820.7	1.14%	1.97%	0.87%	1.43%

**Table:** (a)

	MIP				
	*P*	*H*	*S*	*B*	*T*
A, V	5658.4	6842.5	6645.8	4451.1	23597.8
A, W	5450.5	6214.0	7387.1	4702.6	23754.2
B, V	32158.1	6915.5	13451.7	6877.4	59402.7
B, W	34654.5	5677.2	13542.4	6150.3	60024.4

**Table:** (b)

	*D* = 6				
	*P*	*H*	*S*	*B*	*T*
A, V	7726.2	4102.4	6699.3	5120.8	23648.7
A, W	9451.2	4412.3	5673.1	4672.4	24208.9
B, V	34248.6	5052.0	17536.1	4765.6	61602.4
B, W	35975.2	5735.0	14196.5	5471.9	61378.5

**Table:** (c)

	*D* = 7				
	*P*	*H*	*S*	*B*	*T*
A, V	6314.5	6712.9	6513.1	4731.3	24271.8
A, W	5578.0	6325.3	7392.2	4825.8	24121.3
B, V	32574.5	6937.8	14782.9	6925.6	61220.9
B, W	35112.1	5752.3	13945.9	6184.2	60994.5

*P*: production cost, *H*: holding cost, *S*: setup cost, *B*: purchasing cost, and *T*: total cost.

## Index

*i*: Subassembly *i*
*j*: Disassembly operation *j*.

### Parameter

*T*: The length of planning horizon, *t* = 1, 2, …, *T*
oq_*it*_: Order quantity of subassembly *i* in period *t*
pc_*j*_: Processing cost of operation *j* per unit time
hc_*i*_: Unit holding cost of subassembly *i*
sc_*j*_: Setup cost of operation *j*
pp_*i*_: Unit purchase cost of subassembly *i*
pr_*j*_: Production rate of operation *j* (units/time)
rm_*ij*_: −1 if subassembly *i* is disassembled by operation *j*, the number of subassemblies generated by operation *j*, otherwise
ps_*ij*_: 1 if subassembly *i* is disassembled by operation *j*, 0 otherwise
cs_*ij*_: The number of subassembly *i* produced by operation *j* (∴rm_*ij*_ = cs_*ij*_ − ps_*ij*_)
st_*j*_: Setup time for operation *j*
timeCapa_*t*_: Available work time at period *t*.

### Decision Variables

*X*_*jt*_: Processing time of operation *j* during period *t*
*Y*_*jt*_: 1 if operation *j* is performed during period *t*, 0 otherwise
*Z*_*jt*_: 1 if there is a setup for operation *j* in period *t*, 0 otherwise
Inv_*it*_: Inventory of subassembly *i* in period *t*
Po_*it*_: The number of purchased subassembly *i* in period *t*
*A*_*jt*_: 1 if operation *j* is ready for the first time in period *t*, 0 otherwise
*B*_*jt*_: 1 if operation *j* is ready for the last time in period *t*, 0 otherwise
*W*_*t*_: 1 if there is no operation to be performed in period *t*, 0 otherwise
*δ*_*t*_: 1 if there is more than one operation to be performed in period *t*, 0 otherwise.
